# Review of Materia Medica for the Use of Students

**Published:** 1852-06

**Authors:** 


*Review of Materia Medica, for the use of Students.* By JOHN B. BIDDLE., M. D., formerly Professor of Materia Medica in the Franklin Med. Col. of Philadelphia, Physician to the Pennsylvania Institute for the Deaf and Dumb, Fellow of the College of Physicians of Philadelphia, Member of the Medical Society of Hamburgh. With illustrations. pp. 322, 12mo.: Lindsay & Blakiston, Phil., 1852. From the publishers, through S. C. Griggs & Co.

THIS work is dedicated to medical students throughout the United States. The illustrations of botanical descriptions are restricted to indigenous and naturalized plants. We have a kind of general distrust for abridgements, analyses, and reviews, but the book before us is superior to most of its class. J.

